# Is Performance of Scholars Correlated to Their Research Collaboration Patterns?

**DOI:** 10.3389/fdata.2019.00039

**Published:** 2019-11-05

**Authors:** Hyeon-Ju Jeon, O-Joun Lee, Jason J. Jung

**Affiliations:** ^1^Department of Computer Engineering, Chung-Ang University, Seoul, South Korea; ^2^Future IT Innovation Laboratory, Pohang University of Science and Technology, Pohang-si, South Korea

**Keywords:** bibliographic network embedding, research performance estimation, research group analysis, research collaboration, collaboration pattern discovery

## Abstract

This study aims to validate whether the research performance of scholars correlates with how the scholars work together. Although the most straightforward approaches are centrality measurements or community detection, scholars mostly participate in multiple research groups and have different roles in each group. Thus, we concentrate on the subgraphs of co-authorship networks rooted in each scholar that cover (i) overlapping of the research groups on the scholar and (ii) roles of the scholar in the groups. This study calls the subgraphs “collaboration patterns” and applies subgraph embedding methods to discover and represent the collaboration patterns. Based on embedding the collaboration patterns, we have clustered scholars according to their collaboration styles. Then, we have examined whether scholars in each cluster have similar research performance, using the quantitative indicators. The coherence of the indicators cannot be solid proofs for validating the correlation between collaboration and performance. Nevertheless, the examination for clusters has exhibited that the collaboration patterns can reflect research styles of scholars. This information will enable us to predict the research performance more accurately since the research styles are more consistent and sustainable features of scholars than a few high-impact publications.

## 1. Introduction

As academic societies are getting broader and more subdivided, various intelligent services for scholars have been required (e.g., a recommendation for collaborators, research topics, or journals). For those services, measurements for evaluating performance of scholars, quality of journals, or prominence of research topics are essential and fundamental components.

Therefore, there have been various studies for defining quantitative indicators to evaluate and compare entities in the academia (Hirsch, 2005, 2010; Sidiropoulos et al., 2007; Wu, 2010; Galam, 2011). These indicators have mostly employed (i) count-based and (ii) network-based approaches. The count-based approach comes from intuitive assumptions: a highly-cited scholar/paper/journal might have higher quality than lowly-cited ones, or a scholar published a larger number of papers might have higher performance than the others. However, the assumptions are not “always” correct. First, if a scholar publishes lots of low-quality papers with self-citations, he/she will ostensibly get a lot of highly-cited articles. Also, the number of publications and citations have a dependency on the activeness of research fields. Besides, even if two scholars have the same number of citations, we cannot answer whether the two scholars have similar research performance.

In order to avoid this problem, various indicators have been proposed to evaluate the academic entities based on their influence (i.e., impact in academic communities). They measure the influence of scholars or papers based on bibliographic networks (e.g., co-authorship networks or citation networks). The network-based approaches mostly use centrality measurements to estimate the significance of scholars/papers in the research communities. Nevertheless, estimating the significance is too naïve to reflect what kinds of roles the scholars/papers have in the research communities; e.g., whether a scholar is a principal investigator (PI) of a research group or an independent researcher participating in numerous research projects.

To improve the network-based indicators, various studies (Ganesh et al., 2016; Ganguly and Pudi, 2017) have proposed methods for learning representations of scholars/papers based on structures of the bibliographic networks. However, these methods mostly consider only the first-order proximity for embedding entities in the bibliographic networks. In the case of scholars, the first-order proximity can reflect collaborators of each scholar. Nevertheless, the proximity cannot consider (i) how a group of scholars work together and (ii) what kinds of roles each scholar has in the research group. We assume that characteristics of research groups affect the research of each scholar; not only on the research performance but also on styles of scholars or types of publications. Based on this assumption, we attempt to discover and represent how scholars work together. Then, this pattern of research collaboration might enable us to predict and analyze the performance of the scholars.

Thereby, in this study, we attempt to validate a research question: research collaboration patterns of scholars are correlated to their research performance. To discover and compare the collaboration patterns, we propose a method for learning representations of structural features of co-authorship networks. First, based on subgraph discovery techniques, we extract and describe the collaboration patterns rooted in each scholar. The collaboration patterns are embedded using Word2Vec-based graph embedding methods regarding their scale and adjacency. Finally, we have verified the research question by clustering scholars according to their collaboration patterns. We have examined each cluster for whether scholars in the cluster have coherence in terms of the research performance.

## 2. Related Work

In this section, we introduce the existing approaches for assessing the research performance. And, we also present the existing studies that attempted to validate correlation between collaborations of scholars and their research performance, even though they merely applied centrality measurements to represent the collaborations.

### 2.1. Count-Based Indicators

Papers are a channel that most directly exposes performance of scholars. However, each paper has a different quality, and it is challenging to assess its quality one-by-one. A massive amount of papers are published every year (e.g., 42,311 papers were indexed in DBLP during August 2019), and the papers deal with too diverse research area. To measure the quality of papers, the number of citations is one of the most effective indicators. Therefore, various indicators have been proposed to measure the research performance by considering both the number of citations and papers. Among them, one of the most widely-used indicators is *h*-index (Hirsch, 2005) that considers a ratio of the number of citations for the number of papers. The *h*-index is a more effective method than simply comparing the number of papers and citations, since the *h*-index gives different weights according to quality of papers.

In order to more accurately measure the performance of scholars, other indicators have been proposed to reflect more diverse features of the research performance. First, the *h*-index counts citations of a few top papers. However, it is important to consider overall performance; e.g., *g*-index (Egghe, 2006), *h*_(2)_-index (Kosmulski, 2006), *w*-index (Wu, 2010), *EM*-index (Bihari and Tripathi, 2017), and so on. Second, indicators should reflect that co-authors have different levels of contribution for each paper; e.g., $\bar{h}$-index (Hirsch, 2010), *gh*-index (Galam, 2011), *Ab*-index (Biswal, 2013), and so on. Lastly, recent papers have a relatively smaller number of citations than older ones. Therefore, indicators have to consider publication ages of papers; e.g., *v*-index (Vaidya, 2005), *AR*-index (Jin et al., 2007), contemporary *h*-index (Sidiropoulos et al., 2007), Trendy *h*-index (Sidiropoulos et al., 2007), and so on.

However, most of the count-based indicators only concentrate on results of the research. Measuring the performance based on a part of papers cannot reflect whether the performance is sustainable or not. Co-authorship networks represent not only the performance of scholars but also the way how the scholars collaborate for the results. Thus, the collaboration of a scholar is closer to research capacity, which is an expectation of the performance, than the number of citations or papers. Also, it will enable us to analyze how we can get high research performance. Additionally, the number of citations or papers is also dependent on activeness of research areas. This dependency causes non-interoperability of the quantitative indicators between research areas.

Abbasi et al. (2009) have proposed *RC*-index and *CC*-index for enhancing the count-based indicators by considering quantity of collaborations and quality of collaborators. These indicators evaluate scholars based on their collaboration activities, and the activities are assessed based on citations for co-authored papers. Nevertheless, they only evaluate collaborators of each scholar rather than consider how they work together. The following section introduces indicators for measuring research performance based on collaborations with co-authorship networks in detail.

### 2.2. Network-Based Indicators

Although there have been various studies for analyzing collaborations of scholars, they only concentrated on measuring centrality [e.g., closeness centrality (Sabidussi, 1966), betweenness centrality (Freeman, 1977), PageRank (Haveliwala, 2002), and so on] of each scholar in co-authorship networks. Obviously, the node centrality in social networks indicates how much influence the node has. Nevertheless, these centrality measurements are also affected by the quantitative inequality between research fields. Furthermore, the centrality cannot reflect collaboration styles and organizational cultures of scholars and their research groups. Recently, most of the studies are conducted by collaborations of various-scaled research groups. Therefore, organizations and cultures of the research groups will be key features that affect performance of scholars.

Newman (2001) analyzed structures of co-authorship networks. After this attempt, various studies applied social network analysis techniques on co-authorship networks, mainly focused on the centrality of scholars. Erjia and Ying (2009) validated that centrality of scholars and the number of their citations are significantly related. In their study, betweenness centrality and the number of citations showed the highest correlation. However, both of the measurements can be affected by the number of papers. Therefore, a few studies employed more reasonable indicators to validate the correlation between centrality and performance. A few studies (Yan and Ding, 2011; Waltman and Yan, 2014) validated correlation between PageRank and academic influence of scholars. Ding and Cronin (2011) also attempted to verify that the number of citations for papers cannot reflect influence of scholars on academic societies by measuring PageRank in citation networks. Bordons et al. (2015) showed correlation between centrality of scholars and their *g*-index (Egghe, 2006).

As validated in the existing studies, the network-based indicators are correlated to research performance of scholars. However, methods for estimating performance based on co-authorship networks have been limited to simply measuring the centrality. To detailedly reflect collaboration relationships, a few studies concentrated on that scholars mainly collaborate with a few steady partners. Reyes-Gonzalez et al. (2016) classified scholars into research groups according to frequency of co-authoring. Then, they verified that similar research groups have similar performance. This method is valuable for comparing performance of research groups, not for assessing performance of individual scholars. The existing studies cannot consider that scholars participate in multiple research groups, and members of the groups have different roles and significance. In this perspective, we focus on collaboration patterns in co-authorship networks.

## 3. Representing Collaboration Patterns

This study aims to (i) discover collaboration patterns of scholars and (ii) represent the collaboration patterns. We assume that the collaboration patterns are correlated to research performance of the scholars and implicitly reflect influence of the scholars in academia. First, we propose a method for discovering the collaboration patterns from co-authorship networks, in section 3.1. To detect and describe relationships between each scholar and his/her collaborators, we employ the WL (Weisfeiler-Lehman) relabeling process. Then, to simplify comparisons between the collaboration patterns, we adopt graph embedding techniques. Section 3.2 describes a method for learning representations of the collaboration patterns.

In this paper, we analyze collaborations based on co-authorship networks, which represent the frequency of co-authored publications among scholars. Although there are various kinds of research collaborations (e.g., co-organizing seminars/workshops/conferences, editing journals, planning/operating research projects, and so on) rather than the co-authoring, publications and co-authorships are the most explicit results and forms of collaborations in the research.

**Figure 1:**
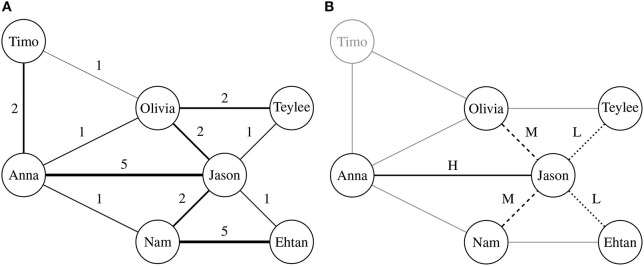
Discovering collaboration patterns from co-authorship networks. In both **(A)** and **(B)**, a node indicates a scholar, and each edge denotes whether two corresponding scholars have ever collaborated. A weight of edge in the co-authorship network indicates proximity between two scholars, which are measured by the number of co-authored publications. **(B)** presents an example of collaboration pattern rooted in “Jason.” To discover collaboration patterns, we classify edges according to proximity distribution of each scholar. Although two scholars share a common collaborator, importance of the collaborator can be different.

As shown in Figure 1A, the co-authorship network is a social network among scholars. In this network, each node indicates a scholar, each edge represents existence of collaborations between two scholars, and a weight on edge is as with frequency of the collaborations between the scholars. Thus, the co-authorship network is an undirected graph. Based on the network, we can analyze how each scholar is connected to other scholars and how each research group works together. The co-authorship network can be defined as:

Definition 1 (Co-Authorship Network). *Suppose that* *n* *is the number of scholars that are in bibliography data. When* N *indicates a co-authorship network*, N *can be described as a symmetric matrix* ∈ ℝ^*n*×*n*^*. Each element of* N *denotes a degree of proximity between two corresponding scholars. This can be formulated as:*

(1) $$\begin{array}{r} N=\left[\begin{array}{ccc} a_{1,1} & \cdots & a_{1,n}\\ \vdots & \ddots & \vdots \\ a_{n,1} & \cdots & a_{n,n} \end{array}\right], \end{array}$$

*where* *a*_*i, j*_ *indicates proximity of* *s*_*i*_ *for* *s*_*j*_ *when* S *is a universal set of scholars that are in bibliography data and* *s*_*i*_ *is the* *i-th element of* S.

In the co-authorship network, relationships between scholars are complicatedly entangled. Graph theory-based measurements can reflect only few aspects of research collaborations (e.g., who are leading research groups). However, to reveal collaboration styles of research groups and scholars, we have to analyze structural features of the research groups and positions of each scholar in the groups. Especially, scholars can participate in multiple research groups at the same time. The existing network-based indicators have difficulty for reflecting various research groups that are overlapped on a scholar.

To deal with this problem, we attempt to extract and describe structures of research groups in multiple scales. The structures are described by collaborators of each scholar on various scales (i.e., *n*-hop connectivity), using subgraph discovery techniques. We assume that the subgraphs of co-authorship networks represent collaboration patterns between scholars. Figure 1B presents an example for extracting a collaboration pattern of “Jason” from the co-authorship network in Figure 1A. The transformation from Figures 1A,B shows reassigning a label rooted in “Jason” based on labels of its collaborators, which only represent one-hop connectivity. This approach has a common point with ego-centered citation networks (Huang et al., 2018), since they commonly concentrate on neighborhoods of a target node in bibliographic networks. Therefore, Figure 1B can be called as “ego-centered co-authorship network.” However, as different from the ego-centered network, we iterate the transformation from Figures 1A,B for each scholar. According to the iteration, coverage of collaboration patterns becomes wider. This approach enables us to represent structures of research groups overlapped on each scholar with various scales. The collaboration pattern is defined as:

Definition 2 (Collaboration Pattern). *Suppose that* $s_{i}^{\left(d\right)}$ *indicates a collaboration pattern of* *s*_*i*_ *at degree* *d* ∈ [0, *D*]*. Collaboration patterns rooted in* *s*_*i*_ *reflect (i) collaborators of* *s*_*i*_ *and (ii) significance of each collaborator for* *s*_*i*_*. Also, the degree lets us know (iii) coverages of the collaboration patterns, which are observation ranges for discovering the patterns. To represent this information iteratively, we describe a collaboration pattern on degree* *d* *based on (i) itself and (ii) its neighborhoods on degree* *d* − 1*. When* *a*_*i, j*_, *a*_*i, k*_, *a*_*i, l*_ *are only non-zero elements within* ∀*a*_*i*, *_, $s_{i}^{\left(d\right)}$ *can be formulated as:*

(2) $$\begin{array}{r} s_{i}^{\left(d\right)}=\langle s_{i}^{\left(d-1\right)};s_{j}^{\left(d-1\right)},s_{k}^{\left(d-1\right)},s_{l}^{\left(d-1\right)}\rangle . \end{array}$$

In the following section, we propose a method for extracting the collaboration patterns from the co-authorship networks.

### 3.1. Discovering Collaboration Patterns

In this study, we extract the collaboration patterns from the co-authorship network using the WL (Weisfeiler-Lehman) relabeling process, which comes from the WL graph isomorphism testing (Shervashidze et al., 2011). The WL relabeling can discover multi-scaled subgraphs rooted in each node by iteratively assigning a new label based on neighbors of the node. The variety of scales lets us know the structures of research groups of each scholar from various viewpoints.

Although the existence of edge provides information about which scholars are connected, collaborations among scholars also have a degree of significance. Considering which collaborators are significant for each scholar will let us know (i) roles of scholars in their research groups and (ii) structures of research groups. Even if a scholar has relationships with multiple other scholars, it does not mean that all the relationships are equivalent. Therefore, the collaboration patterns should be described regarding proximity between scholars. We describe collaboration patterns of scholars based on the (i) adjacency and (ii) distribution of proximity between the scholars. These two features provide the following information.

- Adjacency: The adjacency between scholars in the co-authorship networks indicates that they have collaborated more than one publication.
- Proximity: Among the collaborators, the proximity enables us to discriminate which ones are more significant or valuable collaborators. Also, a case that a few scholars lead most of the studies in a research group is different from another case that all the scholars equally participate in their research. Thereby, the distribution of proximity can reflect even organizational cultures of the research groups.

However, the WL relabeling process cannot consider the degree of proximity (i.e., collaboration frequency), but only the adjacency. To solve this issue, Lee (2019) has proposed a modification of the WL relabeling by labeling edges according to the proximity. We apply this method for discovering collaboration patterns of scholars. Similar to the existing method (Lee, 2019; Lee and Jung, 2019), we classify relationships between scholars into three categories: high ($H_{i}$), medium ($M_{i}$), and low ($L_{i}$) proximity, based on the frequency of collaborations. Nevertheless, research fields and communities have a difference in the amount of collaboration among scholars. Thus, we set adaptive thresholds between the categories according to the distribution of collaboration frequency. When we discover subgraphs rooted in *s*_*i*_, an edge between *s*_*i*_ and *s*_*j*_ (*a*_*i, j*_) can be labeled as:

(3) $$a_{i,j}\in \{\begin{array}{ll} H_{i}, & \text{if }a_{i,j}>\mu_{i}+\theta \cdot \sigma_{i},\\ L_{i}, & \text{else if }a_{i,j}<\mu_{i}-\theta \cdot \sigma_{i},,\\ M_{i}, & \text{otherwise}. \end{array}$$

where μ_*i*_ indicates the average number of collaboration between *s*_*i*_ and his/her collaborators, σ_*i*_ denotes the standard deviation for collaboration frequency of the collaborators, and θ refers to a weighting factor for thresholds between the three categories. Thereby, where $s_{i}^{\left(d\right)}$ indicates a subgraph rooted in *s*_*i*_ at degree *d*, $s_{i}^{\left(d\right)}$ can be described by $s_{i}^{\left(d-1\right)}$ and subgraphs rooted in neighborhoods at degree *d* − 1 in the three categories. This can be formulated as:

(4) $$\begin{array}{r} s_{i}^{\left(d\right)}=\langle s_{i}^{\left(d-1\right)};H_{i}^{\left(d-1\right)},M_{i}^{\left(d-1\right)},L_{i}^{\left(d-1\right)}\rangle , \end{array}$$

(5) $$\begin{array}{r} H_{i}^{\left(d-1\right)}\;=\left\{s_{j}^{\left(d-1\right)}|a_{i,j}\in H_{i}\right\}, \end{array}$$

where $H_{i}^{\left(d-1\right)}$, $M_{i}^{\left(d-1\right)}$, and $L_{i}^{\left(d-1\right)}$ denote sets of subgraphs adjacent with $s_{i}^{\left(d-1\right)}$ in high, medium, and low proximity, respectively. Figure 1B illustrates an example of collaboration pattern, and Algorithm 1 presents all the procedures for discovering the research collaboration patterns, where *N*(*s*_*i*_) indicates a set of collaborators of *s*_*i*_. In Line 2 of Algorithm 1, μ_*i*_ and σ_*i*_ are used for considering which collaborators are more or less significant to *s*_*i*_ than the others. In Line 13, *HASH*(·) indicates the hash function for assigning identifiers for each collaboration pattern.

**Algorithm 1:** Proximity-aware WL relabeling process

1:	**procedure** WLRELABELLING(N,S)
2:	Set $\mu_{i}\leftarrow \frac{1}{\left|N\left(s_{i}\right)\right|}\times \underset{\forall s_{j}\in N\left(s_{i}\right)}{\sum }a_{i,j}$, $\sigma_{i}\leftarrow {\left[\frac{1}{\left|N\left(s_{i}\right)\right|}\times \underset{\forall s_{j}\in N\left(s_{i}\right)}{\sum }{\left(a_{i,j}-\mu_{i}\right)}^{2}\right]}^{\frac{1}{2}}$
3:	**for** *d* : 1 → *D* **do**
4:	Set $H_{i}^{\left(d-1\right)}\leftarrow \emptyset ,M_{i}^{\left(d-1\right)}\leftarrow \emptyset ,L_{i}^{\left(d-1\right)}\leftarrow \emptyset$
5:	**for** *s*_*j*_ ∈ *N*(*s*_*i*_), *s*_*i*_ ≠ *s*_*j*_
6:	$a_{i,j}\leftarrow a_{i,j}\in N$
7:	**if** $a_{i,j}\in H_{i}$ **then**
8:	$H_{i}^{\left(d-1\right)}\leftarrow H_{i}^{\left(d-1\right)}\cup \left\{s_{j}^{\left(d-1\right)}\right\}$
9:	**else if** $a_{i,j}\in M_{i}$ **then**
10:	$M_{i}^{\left(d-1\right)}\leftarrow M_{i}^{\left(d-1\right)}\cup \left\{s_{j}^{\left(d-1\right)}\right\}$
11:	**else**
12:	$L_{i}^{\left(d-1\right)}\leftarrow L_{i}^{\left(d-1\right)}\cup \left\{s_{j}^{\left(d-1\right)}\right\}$
13:	$s_{i}^{\left(d\right)}\leftarrow \langle s_{i}^{\left(d-1\right)};H_{i}^{\left(d-1\right)},M_{i}^{\left(d-1\right)},L_{i}^{\left(d-1\right)}\rangle$
14:	$s_{i}^{\left(d\right)}\leftarrow HASH\left(s_{i}^{\left(d\right)}\right),S\leftarrow S\cup \left\{s_{i}^{\left(d\right)}\right\}$

### 3.2. Learning Representations of Collaboration Patterns

Based on the WL relabeling process, we can describe collaboration patterns of a scholar *s*_*i*_ as a multi-set of subgraphs rooted in *s*_*i*_. To compare collaboration patterns of scholars, one of the most naïve approaches is applying similarity measurements for categorical data (e.g., Jaccard index) to examine whether the scholars have the identical collaboration patterns. However, since the WL relabeling process assigns nominal labels on the collaboration patterns, it is difficult to compare the collaboration patterns by themselves, rather than a composition of them within the scholars.

**Figure 2:**
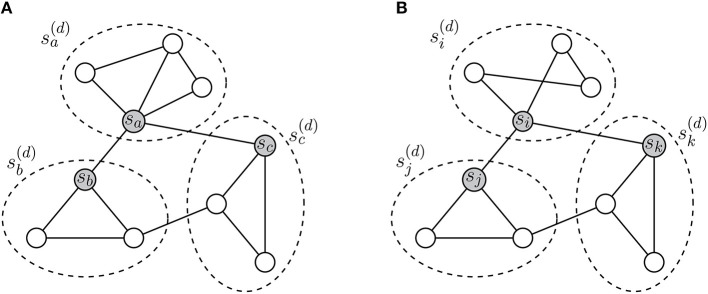
**(A,B)** Learning representations of research collaboration patterns. Dotted ellipses indicate the collaboration patterns rooted in gray nodes. For embedding $s_{a}^{\left(d\right)}$ and $s_{i}^{\left(d\right)}$, collaboration patterns of *s*_*a*_ and *s*_*i*_ have different structures. In the WL relabeling process, labels of the collaboration patterns can provide information only about $s_{a}^{\left(d\right)}\neq s_{i}^{\left(d\right)}$. To compare the collaboration patterns, we attempt to learn representations of the patterns based on their adjacency. Since neighborhoods of *s*_*a*_ and *s*_*i*_ have similar local structures, $s_{a}^{\left(d\right)}$ and $s_{i}^{\left(d\right)}$ are closely located in spite of their structural inequality.

To solve this problem, we propose a method for learning representations of collaboration patterns. Embedding the patterns enables us to easily compare the collaboration of scholars using similarity measurements among vectors. Embedding techniques for entities in graphs (e.g., nodes, subgraphs, meta-paths, and so on) are mostly based on adjacency and proximity between the entities. Although adjacency of subgraphs does not indicate that the corresponding collaboration patterns are similar, vector representations of the subgraphs will reflect their structural features and research groups, including them. Figure 2 presents a simple example of how the adjacency between subgraphs can reach the structural features of the subgraphs.

As shown in (a) and (b) of Figure 2, collaboration patterns are described by adjacency between scholars, and the collaboration patterns also have adjacency with each other. In Figure 2, $s_{a}^{\left(d\right)}$ and $s_{i}^{\left(d\right)}$ have different structures, but neighborhoods of $s_{a}^{\left(d\right)}$ and $s_{i}^{\left(d\right)}$ are structurally identical. When we only apply the WL relabeling, we can obtain information only that $s_{a}^{\left(d\right)}$ and $s_{i}^{\left(d\right)}$ are not identical. Nevertheless, by observing neighborhoods of $s_{a}^{\left(d\right)}$ and $s_{i}^{\left(d\right)}$, we can know that they have structural similarity. In other words, we can identify whether the collaboration patterns have similar meanings. Thus, if we allocate close vector coordinates to adjacent collaboration patterns, $s_{a}^{\left(d\right)}$ and $s_{i}^{\left(d\right)}$ will have similar vector representations, conclusively. Thereby, $\Phi \left(s_{a}^{\left(d\right)}\right)$ and $\Phi \left(s_{i}^{\left(d\right)}\right)$, which are vector representations of $s_{a}^{\left(d\right)}$ and $s_{i}^{\left(d\right)}$, will be able to reflect structural features of research groups including *s*_*a*_ and *s*_*i*_.

We attempt to learn representations of the collaboration patterns using Subgraph2Vec (Narayanan et al., 2016), which is the well-known algorithm based on the adjacency between subgraphs. For embedding, Subgraph2Vec employs radial skip-gram and negative sampling. The radial skip-gram is a modification of the original skip-gram in Word2Vec (Mikolov et al., 2013). In the case of language processing, adjacency of words is determined with fixed window sizes. On the other hand, in the graphical data, such as co-authorship networks, the number of adjacent subgraphs is inconstant. Therefore, the radial skip-gram is used for handling the inconstant number of collaboration patterns with unfixed window sizes. In addition, we compose neighborhoods of *s*_*i*_ on degree *d* based on its adjacent patterns from degree *d* − 1 to *d* + 1, to consider meanings of collaboration patterns on various scales. The negative sampling is applied to reduce the computational complexity in the learning process. Co-occurrence probability of an arbitrary collaboration pattern ($S_{a}$) as a neighborhood of *s*_*i*_ at degree *d* is formulated as:

(6) $$\begin{array}{r} P\left(S_{a}|\Phi \left(s_{i}^{\left(d\right)}\right)\right)≃\sigma \left(\Phi {\left(S_{a}\right)}^{⊺}\Phi \left(s_{i}^{\left(d\right)}\right)\right), \end{array}$$

where σ(·) indicates the sigmoid function, and Φ(·) denotes a projection function for the vector representations.

By modifying the skip-gram and negative sampling (Mikolov et al., 2013), we define an objective function for embedding the collaboration patterns. We maximize the occurrence probability for the neighborhoods and minimize the probability for collaboration patterns that are not neighboring. This is formulated as:

(7) $$\begin{array}{l} L\left(s_{i}^{\left(d\right)}\right)=\sum_{\forall S_{a}\in N\left(s_{i}^{\left(d\right)}\right)}\log\;P\left(S_{a}|\Phi \left(s_{i}^{\left(d\right)}\right)\right)\\ \;-\sum_{\forall S_{b}\notin N\left(s_{i}^{\left(d\right)}\right)}\log\;P\left(S_{b}|\Phi \left(s_{i}^{\left(d\right)}\right)\right)\\ \;≃\sum_{\forall S_{a}\in N\left(s_{i}^{\left(d\right)}\right)}\log\;\sigma \left(\Phi {\left(S_{a}\right)}^{⊺}\Phi \left(s_{i}^{\left(d\right)}\right)\right)\\ \;+\sum_{j=1}^{k}{𝔼}_{S_{b}\sim P_{n}\left(S\right)}\left[\log\sigma \left(-\Phi {\left(S_{b}\right)}^{⊺}\Phi \left(s_{i}^{\left(d\right)}\right)\right)\right], \end{array}$$

where $P_{n}\left(S\right)\propto U{\left(S\right)}^{\frac{3}{4}}$ denotes a noise distribution of collaboration patterns, U(S) refers to a unigram distribution of all the collaboration patterns, and N(·) indicates a set of collaboration patterns that are in neighborhoods. This objective function makes $\Phi \left(S_{a}\right)$ and $\Phi \left(S_{b}\right)$ closer to each other when $S_{a}$ and $S_{b}$ are neighboring. Otherwise, it makes them more distant. We have not significantly modified the objective function and learning methods of Subgraph2Vec. We only have modified and extended the WL-relabeling process to apply Subgraph2Vec on co-authorship networks. The contribution of this study has focused on extracting and comparing the collaboration patterns, but not proposing a novel representation learning method. Therefore, we will not present detail procedures of learning representations to avoid redundancy.

## 4. Evaluation

We have attempted to validate the correlation between the performance of scholars and the research collaboration patterns of scholars. For the validation, we clustered the scholars according to vector representations of their collaboration patterns. Subsequently, we compared the clusters with quantitative indicators for the research performance. Thus, we attempted to examine whether scholars in a cluster exhibit similar research performance. To conduct the comparison, we applied the following indicators: (i) the number of papers written by each scholar, (ii) the total number of citations for all papers written by each scholar, (iii) the average number of citations for all papers written by each scholar, (iv) PageRank (Haveliwala, 2002), (v) betweenness centrality (Freeman, 1977), and (vi) closeness centrality (Sabidussi, 1966). The centrality measurements are calculated for each scholar in the co-authorship network. As a preliminary study, we restrict our observation range into a small part of the bibliographic network. This limitation makes us challenging to measure count-based indicators or acquire the indicators from the external bibliography databases (e.g., Web of Science).

For the experiment, we collected the bibliography data from DBLP dataset^1^ over the last 5 years at the famous conferences (e.g., ICDE, SIGMOD, and VLDB). The dataset consists of rich bibliography information, including the authors, titles, publication year, venues, and so on. The number of citations for the collected papers is acquired from Scopus^2^. Table 1 presents statistical features of the collected dataset. Also, we implemented the proposed model by modifying an open-source project of the Subgraph2Vec^3^. The implemented model has also been publicly accessible^4^. Moreover, the proposed method has various hyper-parameters. We determined the parameters in a heuristic way; the number of epochs (ϵ): 10, the learning rate (η): 0.025, the number of dimensions (δ): 256, the maximum degree (*D*): 3, the number of negative samples (*k*): 200, and the weighting factor (θ): 0.25.

**Table 1:** Descriptions of the experimental dataset.

**Statistics**	**Venues**	**Number of publications**	**Number of scholars**	**Time span**
Value	3	2896	5884	2014–2018

**Figure 3:**
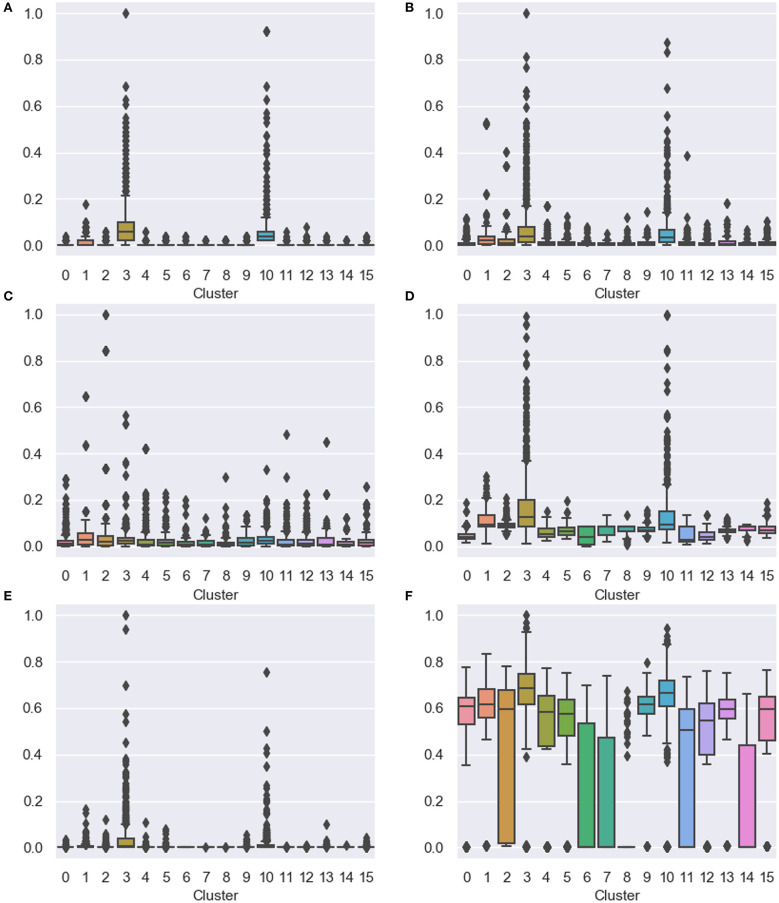
Distribution of the quantitative indicators for scholars in each cluster. Box-and-whisker plots indicate distributions of indicator values, and dots notate outliers. **(A)** The number of papers, **(B)** the total number of citations, **(C)** the average number of citations, **(D)** PageRank, **(E)** betweenness centrality, and **(F)** closeness centrality.

The experimental procedures consist of four steps. First, we extracted collaboration patterns of all the collected scholars based on their adjacency and proximity. Second, we composed vector representations of the scholars by learning representations of the collaboration patterns and concatenating representations of patterns rooted in each scholar. Third, we clustered the scholars based on the vector representations, using the Gaussian Mixture Model and the Expectation-Maximization algorithm. The number of clusters is determined as 16 by minimizing the external adjacency between clusters. Lastly, we analyzed whether scholars in each cluster have a similar research style, based on the quantitative indicators. Table 2 and Figure 3 present the experimental results.

**Table 2:** Experimental results for coherence of the research performance of scholars in each cluster.

		**C#0**	**C#1**	**C#2**	**C#3**	**C#4**	**C#5**	**C#6**	**C#7**	**C#8**	**C#9**	**C#10**	**C#11**	**C#12**	**C#13**	**C#14**	**C#15**
Num	μ	0.14	1.90	0.36	8.70	0.12	0.22	0.11	0.19	0.29	0.40	6.66	0.17	0.17	0.44	0.08	0.31
	σ	0.53	2.42	0.97	**10.33**	0.55	0.67	0.59	0.58	0.70	0.95	**9.98**	0.62	0.64	0.88	0.38	0.80
Sum	μ	0.97	3.59	2.57	6.96	1.17	1.08	0.64	0.68	0.94	1.30	5.58	0.99	0.91	1.59	0.70	1.29
	σ	1.62	7.26	5.74	**10.42**	2.23	1.60	1.13	0.87	1.80	1.82	**8.52**	2.23	1.30	2.86	1.20	1.90
Avg	μ	2.29	4.52	6.02	3.15	2.78	2.40	1.53	1.59	2.05	2.63	3.05	2.13	2.11	3.35	1.65	2.81
	σ	3.96	**7.98**	**14.26**	4.40	5.45	3.41	2.68	2.14	4.20	3.11	2.99	3.75	3.02	6.55	2.74	4.41
PR	μ	4.48	11.18	9.22	16.78	5.70	6.52	4.53	6.88	7.17	7.25	13.18	4.32	4.60	6.80	7.69	6.99
	σ	1.20	4.24	1.98	**13.39**	2.10	1.97	3.77	2.49	2.57	1.76	**11.60**	2.97	2.31	1.56	1.59	2.06
BC	μ	0.03	0.68	0.22	3.83	0.05	0.13	0.00	0.00	0.00	0.26	1.71	0.00	0.00	0.22	0.00	0.13
	σ	0.02	1.87	0.93	**8.37**	0.51	0.74	0.00	0.00	0.00	0.83	**5.36**	0.02	0.06	0.92	0.09	0.47
CC	μ	54.39	58.28	45.34	66.09	46.66	48.20	24.72	17.44	11.42	57.34	63.20	37.44	44.19	55.20	14.48	47.08
	σ	19.04	17.80	**28.35**	16.55	26.36	23.98	**27.50**	26.50	22.44	17.16	17.31	27.38	25.21	19.48	24.17	26.36

*The coherence is indirectly shown by the mean (μ) and standard deviation (σ) of the six quantitative indicators: the number of papers (Num), the total number of citations (Sum), the average number of citations (Avg), PageRank (PR), betweenness centrality (BC), and closeness centrality (CC). Cells present × 10^2^ of μ and σ for the readability. The bold values indicate the first and second highest ones*.

Table 2 presents the mean and standard deviation of each indicator for scholars in a cluster. While most of the clusters had a very low standard deviation, the indicators for two clusters had a much higher standard deviation than the others. Excluding the closeness centrality, clusters which obtained a higher average score from an indicator than the others also had a higher variance for the indicator. This result is caused by that most of the scholars had low performance (e.g., 3870 of 5884 scholars wrote only one paper). At the same time, high-performance scholars exhibited extremely varied values of the indicators, as shown in Figure 3.

Figure 3 presents the distribution of the quantitative indicators for scholars in each cluster using box-and-whisker plots. The box indicates the 1st quartile to the 3rd quartile of distributions of data, and the horizontal bar refers to the 2nd quartile (the median). The ends of the whisker represent the lowest and highest datum within 1.5 interquartile range of the lower and upper quartile. Additionally, we show outliers that refer to data outside the whisker range. The scholars in C#3 and C#10 had the highest variance and the largest number of outliers. Figure 3A presents the scholars in C#3 and C#10 wrote exceptionally more papers than in the other clusters. In our dataset, most of the scholars wrote one or two papers. However, productive scholars wrote a much more number of papers than the others, and there was extremely high variance in the number of papers written by the productive ones. Figure 3B indicates that the scholars in C#3 and C#10 got many citations for their papers. This result can be affected by that the members of C#3 and C#10 had a large number of papers. However, at the same time, their average number of citations is relatively small, as displayed in Figure 3C. Then, we also attempted to examine whether the scholars in C#3 and C#10 had distinctiveness regarding the structure of the co-authorship network. The scholars in C#3 and C#10 are closely connected to other significant scholars, as revealed by the PageRank algorithm in Figure 3D. Also, they had higher betweenness centrality than the others (in Figure 3E). This point indicates that they participated in larger research groups than the others. In Figure 3F, the closeness centrality shows that they directly collaborated with a large number of scholars comparing with scales of their research groups. These results imply that members of C#3 and C#10 might be closely connected and composing large sub-networks.

C#1 and C#2 also showed interesting points. In Figure 3A, the scholars in C#1 and C#2 wrote the small number of papers. On the other hand, in Figure 3B, they had a large number of citations comparing with the number of papers. Especially in Figure 3C, most of the scholars who exhibited the large average number of citations belonged to C#1 and C#2. In other words, the scholars in C#1 and C#2 participated in the small number of papers that obtained a large number of citations. Through these results, we found that they generally concentrated on the quality of papers, not the number of papers. In this perspective, the scholars in C#1 and C#2 had a high performance differently from the scholars in C#1 and C#2. The network-based indicators also showed the difference. As shown in Figure 3E, the members of C#1 and C#2 had a relatively smaller research group than of C#3 and C#10. Although C#3 and C#10 had a similar tendency for all the indicators, C#1 and C#2 showed different results for the PageRank and closeness centrality. In Figure 3F, the scholars in C#1 had many collaborations in their research group. In contrast, the scholars in C#2 looked irrelevant to the direct collaborations, considering a high variance in the closeness centrality. As shown in Figure 3D, the scholars in C#1 had stronger relationships with their collaborators than in C#2.

Furthermore, in most of the indicators, scholars in C#8 obtained low scores, since they wrote only one paper that was infrequently cited. Nevertheless, in Figure 3F, C#8 had many outliers, although most of the other elements had the closeness centrality nearby 0. In other words, most of the scholars in C#8 participated in a paper that had a short author list.

Conclusively, by clustering the collaboration patterns, we have examined whether the collaboration patterns are correlated not only to the performance of scholars but also to their styles of research and collaboration. In both of the cases, the four clusters (C#1, C#2, C#3, and C#10) included scholars who exhibited high performance. However, in terms of the number of publications, the scholars in C#3 and C#10 showed higher performance than in C#1 and C#2. This point is the opposite in terms of the quality of papers. Regarding the structure of research groups, the scholars of C#3 and C#10 had large research groups, they were directly connected to group members, and their collaborators also had high centrality. In C#1 and C#2, the scholars had smaller research groups and fewer adjacent scholars than the former case. While the existing indicators simplify the research performance according to a few features, this result demonstrates that the proposed method can reflect various aspects of the research performance.

## 5. Conclusion

In this study, we have attempted to discover and represent the research collaboration patterns of scholars. Thus, we have proposed a method for learning vector representations of the collaboration patterns rooted in scholars. To demonstrate the efficacy of the method, we clustered the scholars according to the collaboration patterns and compared the clusters with the existing quantitative indicators for the research performance. Based on the comparison, we could partially validate whether the collaboration styles of scholars are correlated to their performance.

The proposed method and evaluation procedures have a few limitations. First, we did not conduct a quantitative evaluation and could not solidly verify the research question. To validate whether collaboration patterns are correlated to the research performance of scholars or not, we should find a way of evaluating their relevancy. Second, although we clustered the scholars, we did not suggest a novel indicator for evaluating the collaboration patterns. We do not know yet which collaboration patterns are helpful for improving research performance. Third, the bibliographic network has time-sequential features that dynamically change. However, since the proposed method does not cover the dynamicity, it considers out-dated publications or collaborations as with recent ones. These limitations should be solved for further research.

## Data Availability Statement

The datasets analyzed in this study can be found in DBLP [https://dblp.uni-trier.de] and Scopus [https://www.scopus.com].

## Author Contributions

H-JJ and O-JL conceived of the presented idea and developed the theory, discussed the results, and contributed to the final manuscript. The experiments were conceived by O-JL and conducted by H-JJ. JJ supervised the findings of this work. All authors reviewed the manuscript. JJ and O-JL provided critical feedback.

### Conflict of Interest

The authors declare that the research was conducted in the absence of any commercial or financial relationships that could be construed as a potential conflict of interest.

## Footnotes

^1^https://dblp.uni-trier.de

^2^https://www.scopus.com

^3^https://github.com/MLDroid/subgraph2vec_gensim

^4^https://github.com/higd963/Collaboration2Vec
